# IL-6 trans-signaling induces plasminogen activator inhibitor-1 from vascular endothelial cells in cytokine release syndrome

**DOI:** 10.1073/pnas.2010229117

**Published:** 2020-08-21

**Authors:** Sujin Kang, Toshio Tanaka, Hitomi Inoue, Chikako Ono, Shoji Hashimoto, Yoshiyuki Kioi, Hisatake Matsumoto, Hiroshi Matsuura, Tsunehiro Matsubara, Kentaro Shimizu, Hiroshi Ogura, Yoshiharu Matsuura, Tadamitsu Kishimoto

**Affiliations:** ^a^Department of Immune Regulation, Immunology Frontier Research Center, Osaka University, Suita, Osaka 565-0871, Japan;; ^b^Medical Affairs Bureau, Osaka Habikino Medical Center, Osaka 583-8588, Japan;; ^c^Department of Molecular Virology, Research Institute for Microbial Diseases, Osaka University, Suita, Osaka 565-0871, Japan;; ^d^Department of Clinical Laboratory, Osaka Habikino Medical Center, Osaka 583-8588, Japan;; ^e^Department of Traumatology and Acute Critical Medicine, Osaka University Graduate School of Medicine, Osaka University, Suita, Osaka 565-0871, Japan

**Keywords:** IL-6, tocilizumab, endothelial cell, cytokine release syndrome, COVID-19

## Abstract

Cytokine release syndrome (CRS) is a life-threatening complication induced by systemic inflammatory responses to infections, including bacteria and chimeric antigen receptor T cell therapy. There are currently no immunotherapies with proven clinical efficacy and understanding of the molecular mechanisms of CRS pathogenesis is limited. Here, we found that patients diagnosed with CRS from sepsis, acute respiratory distress syndrome (ARDS), or burns showed common manifestations: strikingly elevated levels of the four proinflammatory cytokines interleukin (IL)-6, IL-8, monocyte chemotactic protein-1 (MCP-1), and IL-10 and the coagulation cascade activator plasminogen activator inhibitor-1 (PAI-1). Our in vitro data indicate that endothelial IL-6 trans-signaling formed an inflammation circuit for robust IL-6, IL-8, and MCP-1 production and promoted PAI-1 production; additionally, an IL-6 signaling blockade by the human monoclonal antibody tocilizumab blunted endothelial cell activation. Plasma from severe COVID-19 patients similarly exhibited increased IL-6, IL-10, and MCP-1 levels, but these levels were not as high as those in patients with CRS from other causes. In contrast, the PAI-1 levels in COVID-19 patients were as highly elevated as those in patients with bacterial sepsis or ARDS. Tocilizumab treatment decreased the PAI-1 levels and alleviated critical illness in severe COVID-19 patients. Our findings suggest that distinct levels of cytokine production are associated with CRS induced by bacterial infection and COVID-19, but both CRS types are accompanied by endotheliopathy through IL-6 trans-signaling. Thus, the present study highlights the crucial role of IL-6 signaling in endothelial dysfunction during bacterial infection and COVID-19.

## Significance

Cytokine release syndrome (CRS) is a life-threatening complication induced by hyperinflammatory responses. However, no specific immunotherapies are available for its treatment. In this study, we found that interleukin (IL)-6 signaling plays a crucial role in endothelial cell dysfunction during bacterial and viral CRS. Specifically, we identified that the pathogenesis of CRS in patients with sepsis, acute respiratory distress syndrome, and burns involved the IL-6–mediated production of hyperinflammatory cytokines and plasminogen activator inhibitor-1 (PAI-1), which indicates that IL-6 signaling blockade has potential as a therapy for CRS. We also found that the inhibition of IL-6 signaling by tocilizumab treatment decreased PAI-1 production and alleviated clinical manifestations in severe COVID-19 patients.

Cytokine release syndrome (CRS) refers to the state in which various cytokines are extremely elevated through excessive immune responses to bacterial or viral infections or tissue injuries. CRS is accompanied by different conditions, including systemic inflammatory response syndrome, hemophagocytic lymphohistiocytosis (HLH), macrophage activation syndrome (MAS), chimeric antigen receptor (CAR) T cell engaging therapy, and sudden acute respiratory syndrome coronavirus 2 (SARS-CoV-2) infection (COVID-19) (1, 2). CRS is a harmful systemic hyperactivated immune state that can lead to vascular leakage, transaminitis, coagulopathy, multiple organ dysfunction, and death (3). Various cytokines, such as interleukin (IL)-6, IL-10, interferon (IFN)-γ, monocyte chemotactic protein-1 (MCP-1), granulocyte-macrophage colony-stimulating factor, tumor necrosis factor (TNF)-α, IL-2, and IL-8, are released during CRS (4, 5). Despite accumulating information regarding CRS pathophysiology, general supportive care remains the pillar of treatment because a single therapeutic target for immunomodulation is ineffective (6, 7).

IL-6 is a proinflammatory cytokine with pleiotropic functions (8). It mainly signals through both classic-signaling and trans-signaling pathways, and the distinction is dependent on the expression patterns of the IL-6 receptor (IL-6R) and gp130 on the surface of cells, such as immune and endothelial cells, respectively (9). The IL-6R antagonist tocilizumab suppresses both classic-signaling and trans-signaling pathways (8). Recently, tocilizumab resolved HLH-like manifestations in CRS patients caused by CAR T cell therapy and viral infections, such as SARS-CoV-2 infection (10, 11). Notably, the pandemic of COVID-19 caused by infection with the novel coronavirus SARS-CoV-2 is ongoing worldwide, and this disease has a high mortality rate of ∼5% (12). However, no effective therapies have been found yet, and because the pathogenesis of severe COVID-19 is still poorly understood, many physicians treat severe COVID-19 patients by following the sepsis treatment guidelines (12, 13). Clinically, sepsis is associated with tissue edema caused by vascular leakage through damaged endothelial cells. Endothelial dysfunction is the principal determinant of microvascular dysfunction, as it shifts the vascular equilibrium toward more vasoconstriction, causing subsequent organ ischemia, systemic inflammation with associated tissue edema, and a procoagulant state (14). However, it remains to be clarified whether the activation of IL-6R signaling affects endothelial cells during CRS caused by bacterial or viral sepsis. In this study, we investigated the role of IL-6 signaling in the regulation of endothelial activation and its involvement in the pathogenesis of CRS.

## Results

### All Patients with CRS Have Common Manifestations in Cytokine Levels.

**Fig. 1.**
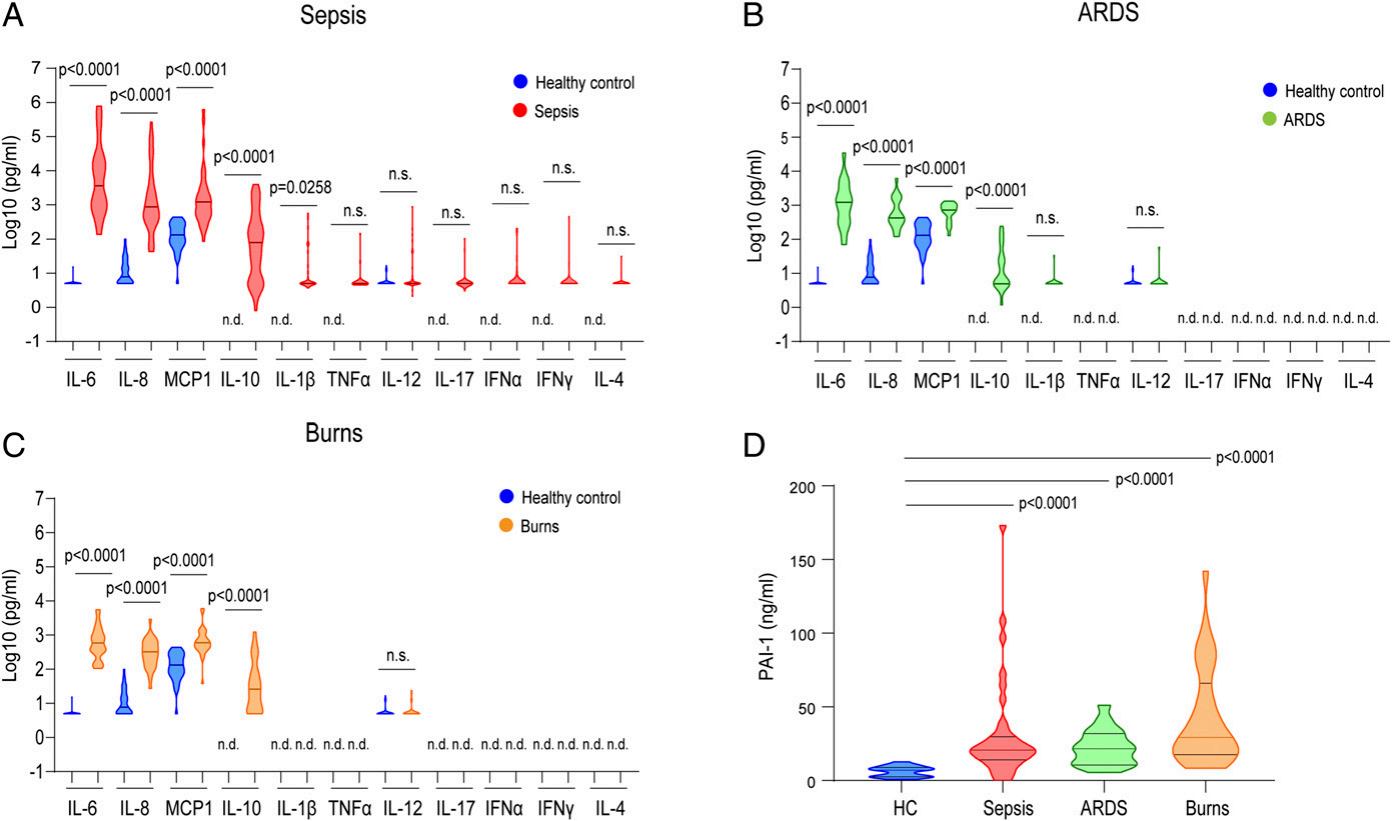
Cytokine and chemokine profiles of CRS patients. (*A*‒*D*) Cytokine, chemokine (*A*‒*C*), and PAI-1 (*D*) levels were measured in the sera of patients with CRS (sepsis, *n* = 37; ARDS, *n* = 19; burns, *n* = 35). Comparisons were made by using a Mann‒Whitney *U* test (*A*‒*C*) or Kruskal‒Wallis test for multiple-comparisons (*D*). Statistically significant differences are indicated; n.s.: nonsignificant; n.d.: nondetectable. Bar indicates the median. Blue graph indicates healthy control (HC, *n* = 36). Other colors are indicated in the graph.

Elevated levels of proinflammatory cytokines are associated with the clinical manifestations of CRS (15, 16). For example, several cytokines in the sera of sepsis or burn patients are correlated with patient prognosis and disease severity (17, 18). To explore the importance of the cytokine network in the pathogenesis of CRS, we reconstituted and reanalyzed the cytokine profiles of sera from CRS patients (17, 18). A total of 91 patients with CRS associated with sepsis, acute respiratory distress syndrome (ARDS), or burns were enrolled in this study. The clinical characteristics of these patients are summarized in *SI Appendix*, Table S1. Serum concentrations of 11 cytokines and chemokines were measured in healthy controls (*n* = 36), patients with sepsis (*n* = 37), patients with ARDS (*n* = 19), and patients with burns (*n* = 35; Fig. 1 *A*‒*C*). In the sepsis patients, the serum levels of the proinflammatory cytokines IL-6, IL-8, MCP-1, and IL-10 were strikingly higher than those in healthy controls, and the serum levels of IL-1β were slightly elevated (Fig. 1*A*). In contrast, the levels of TNF-α, IL-12p40, IFN-α, IFN-γ, IL-17, and IL-4 in the sepsis patients were not significantly different from those in the healthy controls (Fig. 1*A*). To a lesser extent, the patients with ARDS or burns exhibited higher levels of IL-6, IL-8, MCP-1, and IL-10 compared with the healthy controls (Fig. 1 *B* and *C*). These results indicate that IL-6, IL-8, MCP-1, and IL-10 were commonly elevated in the patients with CRS.

The clinical severity of systemic inflammation is associated with endothelial injury and coagulopathy through the induction of vascular leakage and tissue hypoxia, which results in hypotension and multiple organ dysfunction. Elevated PAI-1 levels exacerbate the progression of systemic inflammation, especially sepsis-induced disseminated intravascular coagulation (19). To investigate the pathological implications of PAI-1 levels in systemic inflammation, we measured the serum concentrations of PAI-1 in patients with CRS. As shown in Fig. 1*D*, the serum PAI-1 levels were strikingly elevated in CRS patients as compared with healthy controls. Notably, the serum PAI-1 levels were not elevated in patients with chronic diseases such as rheumatoid arthritis or other connective tissue diseases, whereas levels of 11 cytokines and chemokines were increased (*SI Appendix*, Fig. S1 *A* and *B*). These data indicate that the concentration of PAI-1 is a predictor of CRS disease progression.

### Correlation of Serum IL-6 Levels with Other Cytokine and PAI-1 Levels.

**Fig. 2.**
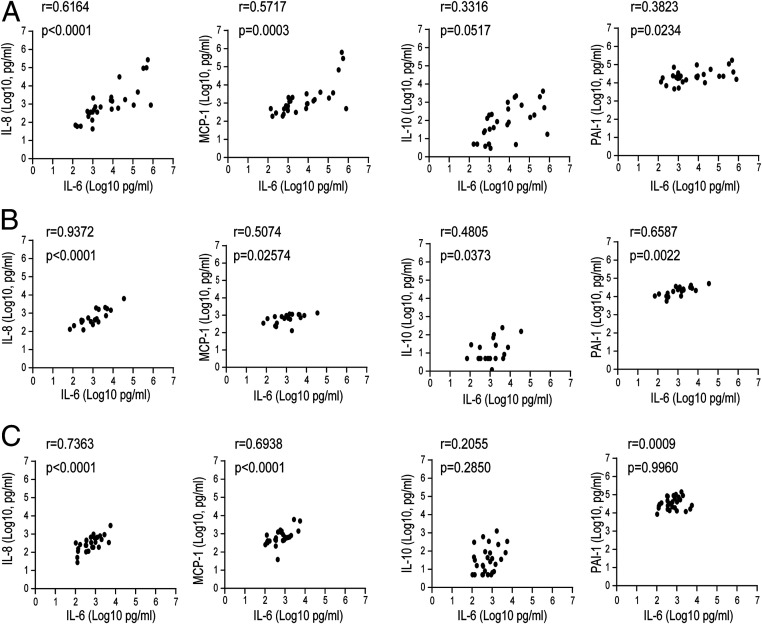
Correlation between IL-6 levels and cytokines and chemokines in patients with CRS. (*A*–*C*) Pearson’s correlations between cytokine and PAI-1 levels in each cohort of CRS patients: sepsis (*A*), ARDS (*B*), and burns (*C*). The *r* coefficient of correlation (from Pearson’s correlation of determination) and the respective *P* values are shown.

Although TNF-α and IL-1β play major roles in the pathogenesis of sepsis in murine models, these cytokines have limited use as clinical markers for at-risk patients (20). Among the proinflammatory cytokines, IL-6 is the most clinically suitable biomarker for sepsis (21). To determine the clinical implications of IL-6 levels for CRS severity, we examined the correlations between serum levels of IL-6 and those of other cytokines as well as of PAI-1. The relative levels of IL-6 were positively associated with those of IL-8, MCP-1, and PAI-1 (*r* = 0.6164, *P* < 0.0001; *r* = 0.5717, *P* = 0.0003; and *r* = 0.3823, *P* = 0.0234, respectively) in the sera of sepsis patients (Fig. 2*A*). In ARDS patients, the IL-6 serum levels showed significant positive correlations with the serum levels of endothelial injury indicators such as IL-8, MCP-1, and PAI-1 (*r* = 0.9372, *P* < 0.0001; *r* = 0.5074, *P* = 0.02574; and *r* = 0.6587, *P* = 0.0022, respectively; Fig. 2*B*). We also found similar correlations between the serum levels of IL-6 and those of IL-8 and MCP-1 in burn patients, whereas serum levels of IL-6 and PAI-1 were not significantly correlated in these patients (Fig. 2*C*). The correlation pattern of the three cohort CRS patients was different, but serum IL-6 levels were positively correlated with elevated IL-8, IL-10, MCP-1, and PAI-1 levels in all CRS patients, which suggests that a common pathological pathway was responsible for the expression of these cytokines during CRS (Fig. 2 *A*‒*C*). The correlations between all cytokines in CRS patients are summarized in *SI Appendix*, Fig. S2.

### IL-6R Trans-Signaling Forms an Inflammatory Circuit in Endothelial Cells.

**Fig. 3.**
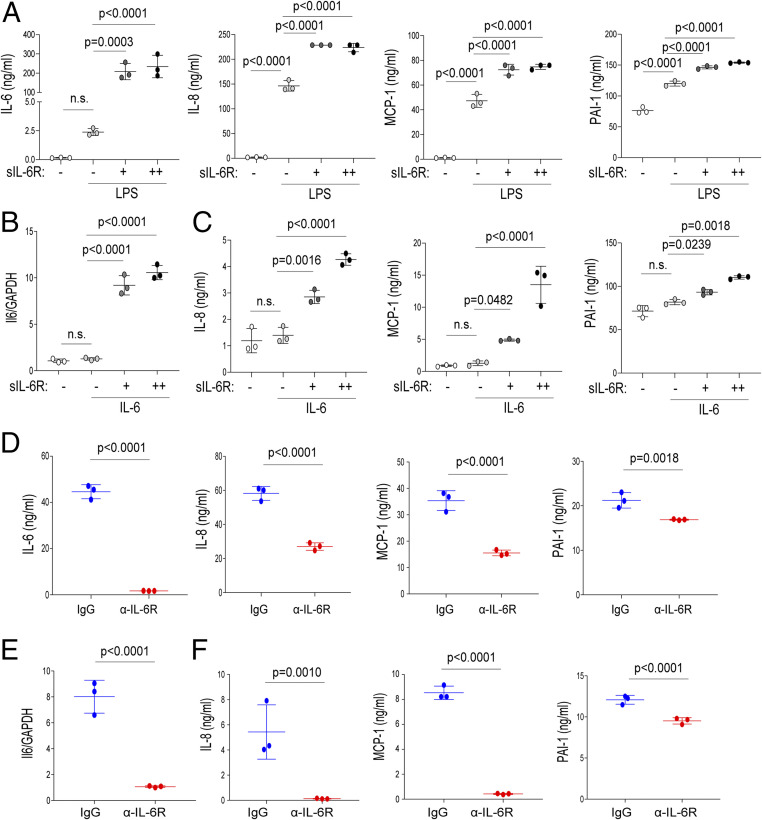
Effect of IL-6R trans-signaling inhibition on cytokine release from endothelial cells. (*A*, *C*, *D*, and *F*) HUVECs were treated with LPS (100 ng/mL) or IL-6 (20 ng/mL) in the presence of sIL-6R (+: 50 ng/mL, ++: 100 ng/mL) in vitro. IL-6, IL-8, MCP-1, and PAI-1 levels in the culture supernatants after 72 h are shown. (*B* and *E*) HUVECs were treated with IL-6 in the presence of sIL-6R. IL-6 mRNA levels after 6 h are shown. Antibodies (20 μg/mL) were applied at the same time as treatment with LPS or IL-6. Statistical comparisons are indicated; n.s.: nonsignificant. The *P* values were determined using an unpaired two-tailed Student’s *t* test (*D*–*F*) or one-way ANOVA (*A*–*C*). Data are representative of three independent experimental replicates and presented as means ± SD. *n* = 3 samples per group in *A*–*F*.

Numerous studies have indicated a relationship between coagulopathy and systemic inflammation. Based on these reports, we next investigated which cell types are involved in the IL-6–mediated production of inflammatory cytokines and PAI-1 during CRS. Endothelial cells express gp130 but not transmembrane IL-6R and produce IL-6 and MCP-1 upon several stimuli. Additionally, endothelial cells express Toll-like receptor 4 (TLR4), which recognizes pattern- and damage-associated molecular patterns. Engagement of TLR4 causes robust IL-6 production in endothelial cells (22). Next, we investigated the response of human umbilical vein endothelial cells (HUVECs) to lipopolysaccharide (LPS) alone or in combination with soluble IL-6R (sIL-6R) for the release of proinflammatory cytokines and PAI-1. We found that LPS and sIL-6R treatment induced a prominent increase in IL-6, IL-8, MCP-1, and PAI-1 production in an sIL-6R dose-dependent manner, whereas LPS alone induced these factors but to a lesser extent (Fig. 3*A*). In this experiment other cytokines, including IL-10, TNF-α, IL-1β, IFN-α, and IFN-γ, were not detectable (*SI Appendix*, Fig. S3*B*). These results indicate that IL-6R trans-signaling is critical for production of several cytokines and of PAI-1. We also found that the treatment of HUVECs with IL-6 in combination with sIL-6R enhanced IL-6 messenger RNA (mRNA) expression (Fig. 3*B*) as well as the release of IL-8, MCP-1, and PAI-1 (Fig. 3*C*), whereas treatment with IL-6 alone had a very limited effect. These findings indicate that IL-6 requires sIL-6R to induce the production of PAI-1 and proinflammatory cytokine in HUVECs.

Because TNF-α and IL-1β activate endothelial cells during sepsis (23), we investigated whether other cytokines could similarly induce proinflammatory cytokine and PAI-1 production in endothelial cells. As shown in *SI Appendix*, Fig. S3*A*, TNFα treatment induced MCP-1 and PAI-1 production but not IL-6 or IL-8 production in HUVECs, whereas stimulation with IL-1β induced the production of IL-8, MCP-1, and PAI-1, as well as that of IL-6 to a lesser extent. In contrast, stimulation with IL-8 or MCP-1 alone did not induce the release of cytokines or PAI-1 in HUVECs.

To further elucidate the pathways regulating inflammatory responses and coagulation activation by IL-6 trans-signaling, we treated HUVECs with the pharmacological IL-6 receptor inhibitor tocilizumab and assessed the cytokine and PAI-1 expression after stimulating the cells with LPS or IL-6 in the presence of sIL-6R. Consistent with the results described in a previous report (24), we found that the treatment of HUVECs with tocilizumab prevented the trans-signaling–induced expression of IL-6, IL-8, MCP-1, and PAI-1 (Fig. 3 *D* and *E*). Overall, these results indicate that IL-6 trans-signaling forms an inflammatory circuit in the endothelium, and a blockade of this signaling prevents PAI-1 and proinflammatory cytokine production.

### Blockade of IL-6 Signaling Decreases Serum PAI-1 Levels in Severe COVID-19 Patients.

We next assessed immune activation and dysregulation in patients with severe SARS-CoV-2 infection. The characteristic features and clinical courses of severe COVID-19 patients enrolled in the present study are shown in *SI Appendix*, Table S2. These patients included one patient who was critically ill and required artificial ventilator management before they could be given a tocilizumab injection, two patients who rapidly progressed to respiratory failure within 2 d after tocilizumab treatment, and four patients who required oxygen inhalation before they could be given a tocilizumab injection. All seven patients recovered promptly from fever and malaise and displayed lower oxygen intake after receiving tocilizumab treatment.

**Fig. 4.**
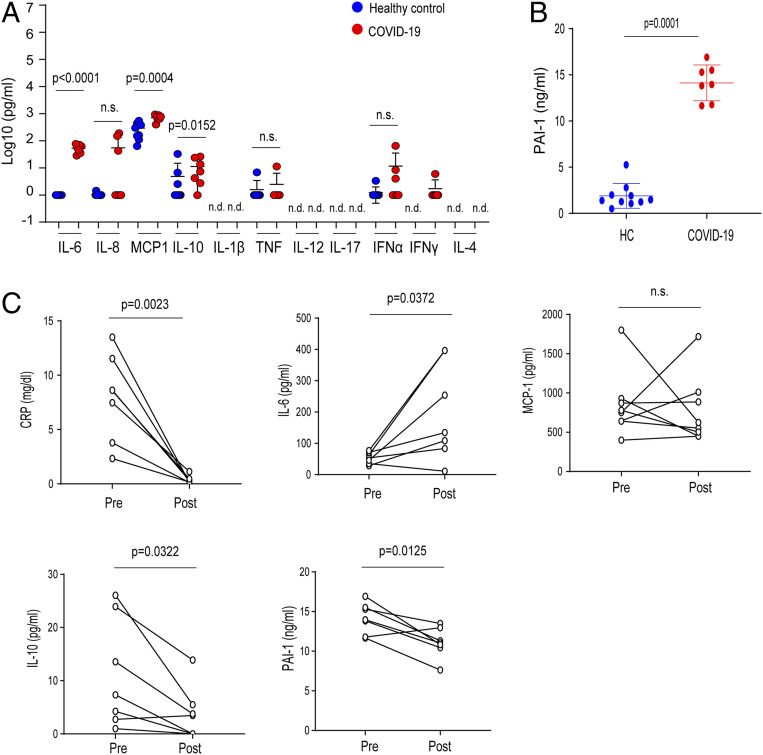
Effect of disrupting IL-6 signaling on PAI-1 production in severe COVID-19 patients. (*A* and *B*) Levels of 11 cytokine and chemokines (*A*) and PAI-1 (*B*) in the sera of severe COVID-19 patients. Blue indicates healthy control (HC, *n* = 10). Red indicates severe COVID-19 (*n* = 7). (*C*) Changes in cytokines, CRP, and PAI-1 levels in seven patients before (pre) and after (post) treatment with tocilizumab. Comparisons were made by applying a Mann‒Whitney *U* test followed by correction (*A* and *B*) or a paired two-tailed *t* test (*C*). Statistical comparisons are indicated; n.s.: nonsignificant.

To investigate the pathogenesis of SARS-CoV-2 infection, we measured the circulating concentrations of 11 cytokines and chemokines and PAI-1 in COVID-19 patients before treating them with tocilizumab. Similar to our findings in patients with bacterial CRS, the serum concentrations of IL-6, MCP-1, and IL-10 in all patients with severe COVID-19 were significantly elevated compared with those in healthy controls (Fig. 4*A*). The serum levels of IL-6, MCP-1, and IL-10 in the severe COVID-19 patients were all relatively lower than the corresponding levels in our three cohorts of CRS patients (sepsis, ARDS, and burns). Although the circulating IL-8 concentration trended higher in the seven patients with severe COVID-19 compared with the healthy controls, the difference failed to reach statistical significance (Fig. 4*A*). Several other cytokines, including IL-1β, IL-12p40, and IL-17, were not detectable in the severe COVID-19 patients. Notably, the severe COVID-19 patients who exhibited severe respiratory dysfunction had significantly higher levels of PAI-1 to a similar extent as those in patients with sepsis, ARDS, and burns (Figs. 4*B* and 1*D*). These results indicate that, as in cases of sepsis and ARDS, a progression of endothelial cell injury occurs through elevated PAI-1 levels in cases of severe COVID-19. After tocilizumab treatment, the seven severe COVID-19 patients exhibited decreased serum PAI-1 levels and improved clinical features, including lower C-reactive protein (CRP) levels. Thus, IL-6 signaling regulated PAI-1 production in severe COVID-19 patients (Fig. 4*B* and *SI Appendix*, Table S2). Furthermore, treatment with tocilizumab decreased the serum concentrations of IL-10, whereas the serum levels of MCP-1 was unaffected (Fig. 4*B*). Because tocilizumab prevents the binding of IL-6 to IL-6 receptors, thereby increasing the serum concentration of free IL-6 (25), it is not surprising that the serum IL-6 concentration in the severe COVID-19 patients was increased after these patients were treated with tocilizumab.

## Discussion

In this study, we found that elevations in IL-6, IL-8, IL-10, MCP-1, and PAI-1 serum levels were common features in patients with sepsis, ARDS, or burns and that the serum IL-6 level was positively correlated with the serum levels of other cytokines and of PAI-1 in CRS patients. The features of COVID-19–mediated CRS are distinct from those of bacterial CRS: 1) COVID-19 patients have a relatively lower elevation in the serum levels of inflammatory cytokines, including IL-6, IL-10, and MCP-1 and 2) these patients have a similar elevation of PAI-1 serum levels, which can be decreased by the inhibition of IL-6 signaling. The present findings suggest that IL-6 signaling plays a central role in the production of these cytokines and of PAI-1 during CRS pathogenesis.

Previous studies have reported that the elevation in IL-6, IL-10, and IFN-γ levels in CRS, including CAR T cell therapy and severe COVID-19, is a typical phenomenon in HLH/MAS, wherein activated macrophages or T cells are the major producers of proinflammatory cytokines (26, 27). Consistently, our clinical data showed that IL-6 and IL-10 levels were significantly elevated in all enrolled CRS patients. These elevations may reflect macrophage activation. Our in vitro data indicate that IL-6 trans-signaling augmented IL-6, IL-8, MCP-1, and PAI-1 production and was blocked by treatment with tocilizumab in HUVECs. IL-6 generally activates the JAK/STAT3 pathway regardless of whether it binds to the soluble or membrane type of IL-6R through gp130 (8). The IL-6‒STAT3‒nuclear factor κB pathway augments *IL-6* gene expression and partially induces MCP-1 and IL-8 expression in vascular endothelial cells (28). However, the precise mechanisms by which IL-6 trans-signaling drives the expression of IL-6, MCP-1, IL-8, and PAI-1 genes remain to be clarified.

The proinflammatory cytokine IL-6 is critical for the progression of acute inflammatory diseases such as sepsis and ARDS. Hyperinflammation in ARDS is characterized by an excessive increase in cytokines, which is considered a CRS. Consistent with previous reports, the serum IL-6 concentrations in the severe COVID-19 patients examined in this study were relatively lower than those in patients with severe ARDS (29, 30). Notably, the significant elevation in PAI-1 levels in patients with severe COVID-19, which is comparable to that observed in patients with ARDS, indicates the induction of vascular endothelial damages in these patients. Additionally, SARS-CoV-2 directly infects vascular endothelial cells, inducing endotheliitis, which indicates vascular endothelial damage occurs in patients with COVID-19 (31). Given the poor clinical outcomes of severe COVID-19 patients despite their relatively lower levels of IL-6, it is noteworthy that COVID-19 ARDS patients exhibit severe endotheliopathy in the lungs, including the presence of microthrombi (32).

Our data indicate that elevated PAI-1 levels were correlated with IL-6 levels in CRS and that they were induced by endothelial cells through IL-6 trans-signaling. Notably, the decrease in PAI-1 levels and clinical improvements exhibited by COVID-19 patients following tocilizumab injection suggest that tocilizumab treatment improved the vascular endothelial functions in these patients. Collectively, our findings suggest the possibility that elevated PAI-1 levels support the progression of endotheliopathy and coagulopathy in severe COVID-19 patients. However, the present work has some limitations in its ability to explain how IL-6 levels are linked to PAI-1 production.

In conclusion, our findings indicate that the serum IL-6 level is positively correlated with the serum levels of PAI-1 in patients with CRS, including those with sepsis or ARDS. This suggests that IL-6 trans-signaling forms a positive feedback loop and plays pivotal roles in endothelial cell injury and coagulopathy, thereby mediating systemic inflammation and deteriorating systemic circulation. Additionally, the IL-6 trans-signaling‒PAI-1 axis is also critical for the pathogenesis of COVID-19–induced CRS, which leads to endotheliopathy and coagulopathy. Collectively, this work provides insights into endothelial activation by IL-6 trans-signaling and our findings may reveal new therapeutic opportunities for the treatment of CRS.

## Materials and Methods

### Patients.

Adult patients with several diseases were recruited for this study. The diagnosis was made according to the relevant diagnostic or classification criteria. The cohort included healthy controls (*n* = 36) and patients with sepsis (*n* = 37), ARDS (*n* = 19), or burns (*n* = 35). The study was approved by the local research ethics committee of each institution (Osaka University Hospital permit number 16109 and Chukyo Hospital permit number 2014015). Informed consent was obtained from patients or their relatives and healthy volunteers for the collection of all blood samples. Seven patients with severe-to-critical COVID-19 were intravenously administered 400 mg tocilizumab once in combination with anti–SARS-CoV-2 drugs. The off-label compassionate use of tocilizumab was approved by the Ethics Committee of Osaka Habikino Medical Center (approval ID 150-7).

### Cell Cultivation.

HUVECs (Lonza) were maintained in EGM-2 medium supplemented with 2% fetal bovine serum (Lonza) and cultured in a humidified incubator at 37 °C with 5% CO_2_. For cytokine stimulation, HUVECs were seeded in 24-well plates at 20,000 or 50,000 cells per well.

### Agents.

Recombinant human IL-6 (R&D Systems), soluble IL-6R (R&D Systems), and Ultrapure LPS (InvivoGen) were used for HUVEC cultivation. Tocilizumab was purchased from Chugai Pharmaceutical Co. Ltd.

### RNA Isolation and qRT-PCR.

Total RNA was isolated from HUVECs using an RNeasy Mini Kit (Qiagen K.K.). Complementary DNA was synthesized using a PrimeScript RT reagent Kit (Takara Bio). A Quant Studio 3 real-time PCR instrument (Applied Biosystems) was used for qPCR with a 2×PCR Master Mix (Applied Biosystems) and primers specific for *IL-6* (Hs00174131_m1) and *β-actin* (Hs01060665_g1) according to the manufacturer’s instructions. qPCR was performed at 95 °C for 15 s followed by 60 °C for 1 min for 40 cycles. The IL-6 mRNA expression level was normalized to the level of β-actin mRNA. mRNA levels of each gene were analyzed using the ∆∆ Ct method against the corresponding mRNA level of the housekeeping gene β-actin.

### Measurement of Cytokines and PAI-1.

IL-1β, IL-4, IL-6, IL-8, IL-10, IL-17A, IL-12/23p40, IFN-α, IFN-γ, MCP-1, and TNF-α levels in sera or appropriately diluted sera were measured using a cytometric bead array kit (BD Biosciences) and a FACS Cant II flow cytometer (BD Biosciences). The detection range of the assay for each cytokine was 10 to 2,500 pg/mL. The serum PAI-1 level was measured using a quantitative ELISA kit (R&D Systems). The detection range of the human PAI-1 assay was 70 pg/mL to 5 ng/mL.

### Statistical Analysis.

Qualitative data were presented as the mean and SD and compared using unpaired *t* tests or the mean and SE and compared using the Mann‒Whitney *U* test for small groups. Paired comparisons were performed using the *t* test. Parametric correlations were made according to Pearson’s correlation. *P* < 0.05 was considered significant.

## Supplementary Material

Supplementary File

## Data Availability

All data are included in the paper and *SI Appendix*.
