# Supplementary material for: IL-6 trans-signaling induces plasminogen activator inhibitor-1 from vascular endothelial cells in cytokine release syndrome
Source: Proc Natl Acad Sci U S A. 2020 Aug 21;117(36):22351–6. doi: 10.1073/pnas.2010229117 (PMC7486751; doi:10.1073/pnas.2010229117)
Supplement: Supplementary File [file pnas.2010229117.sapp.pdf]

PNAS
www.pnas.org

Supplementary Information for

IL-6 trans-signaling induces plasminogen activator inhibitor-1 from vascular endothelial cells in cytokine release syndrome

Sujin Kang, Toshio Tanaka, Hitomi Inoue, Chikako Ono, Shoji Hashimoto, Yoshiyuki Kioi, Hisatake Matsumoto, Hiroshi Matsuura, Tsunehiro Matsubara, Kentaro Shimizu, Hiroshi Ogura, Yoshiharu Matsuura, Tadamitsu Kishimoto

Corresponding author: Tadamitsu Kishimoto
Email: kishimoto@ifrec.osaka-u.ac.jp

This PDF file includes:

Figures S1 to S3
Tables S1, S2

www.pnas.org/cgi/doi/10.1073/pnas.2010229117

Figure S1

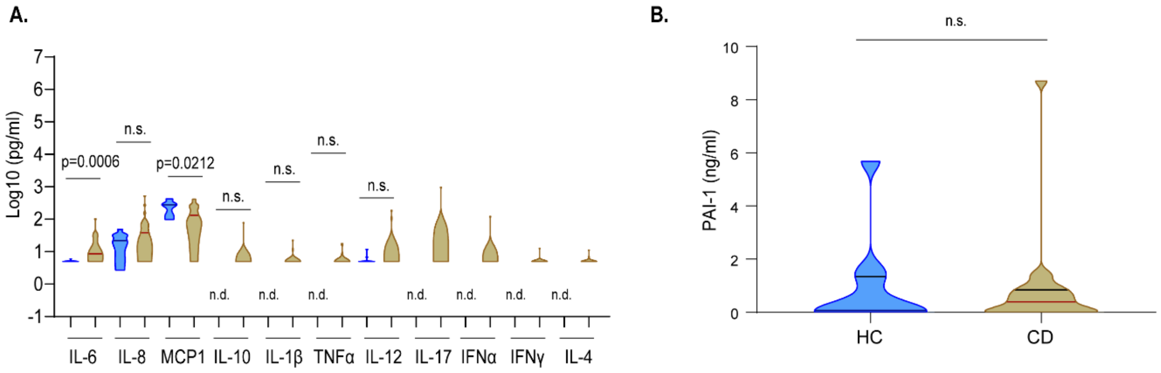

Fig. S1. Cytokine and chemokine profiles of chronic disease patients. Nineteen patients with chronic diseases incused 8 with rheumatoid arthritis, two each with polymyositis/dermatomyositis, systemic sclerosis, and polymyalgia rheumatica, and one each with adult-onset Still disease, Behçet disease, systemic lupus erythematosus, IgG4-related disease, and mixed connective tissue disease, and healthy control (n=10) (A, B) Cytokine, chemokine (A) and PAI-1 (B) levels were measured in sera of patients with cytokine release syndrome. Comparisons were made by the Mann-Whitney U-test followed by correction (A), and the unpaired two-tailed *t*-test (B). Statistical comparisons are indicated, ns: non-significant; nd: non-detectable. Bar indicates the median.

Figure S2

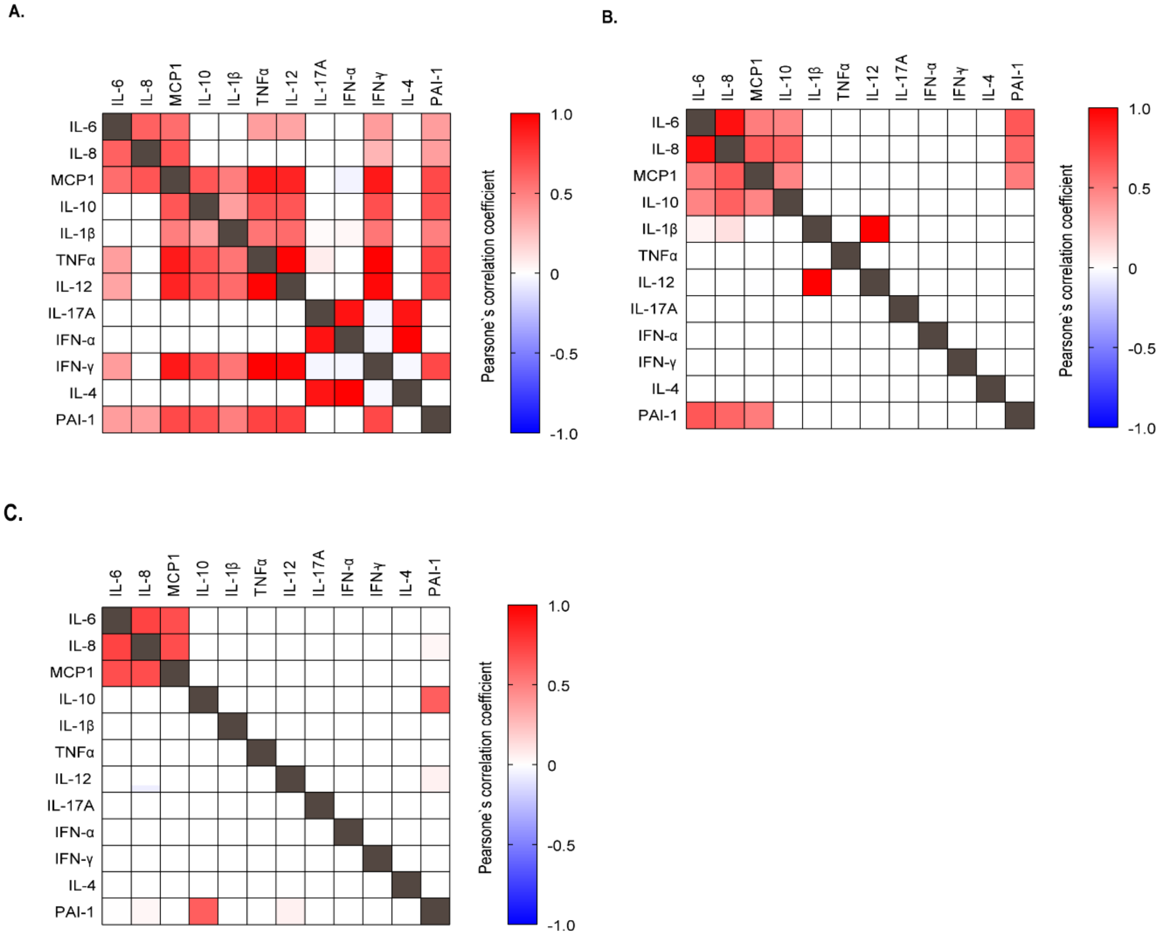

Fig. S2. Pearson's correlation of each cytokine in CRS patients. Hierarchical clustering of Pearson's correlations between cytokines and PAI-1 in patients with each cytokine release syndrome (Sepsis, A; ARDS, B; burns, C). *r*, Pearson correlation of determination. All data with $p<0.05$ was shown.

Figure S3

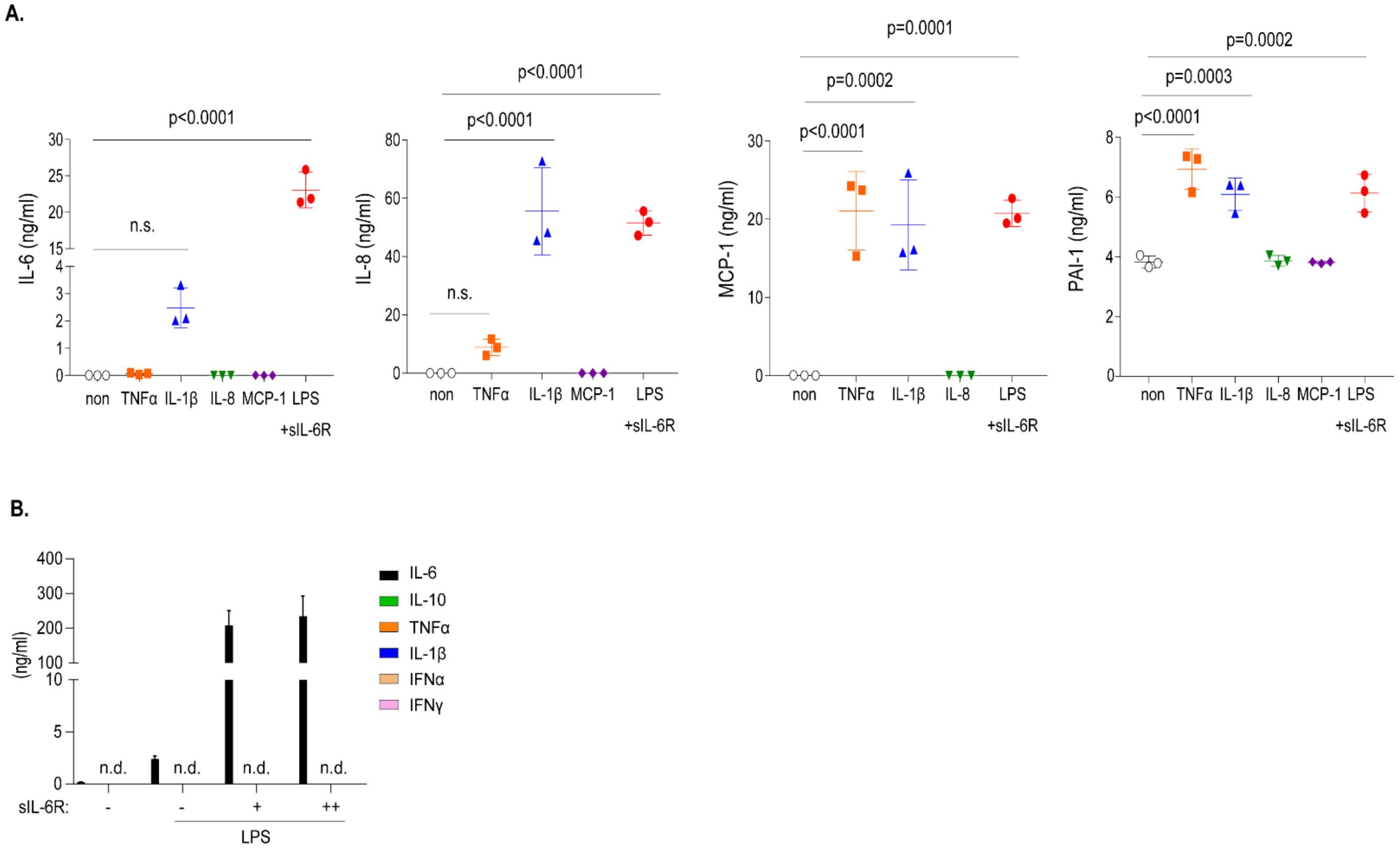

Fig. S3. Responses of HUVECs by stimulation of several cytokines or LPS with sIL-6R. HUVECs were treated with TNFα (10 ng/ml), IL-1β (5 ng/ml), MCP-1 (100 ng/ml), IL-8 (50 ng/ml) LPS and sIL-6R. Levels of IL-6, IL-8, MCP-1 and PAI-1 in the culture supernatants after 48 h are shown. n.s., not significant. *P*-values were determined by unpaired one-way ANOVA (A). The indicated cytokines in the supernatant of HUVECs of Fig. 3A were detected by ELISA (B). Statistical comparisons are indicated, ns: non-significant; nd: non-detectable. Data are representative of three independent experimental replicates, and are presented as means ± SD [n = 3 samples per group (A)].

TABLE S1

**Healthy control (n=36)**	
Male gender (n, %)	35/3 (88%)
**Sepsis (n=37)**	
Age	67 (53–78)
Male gender (n, %)	25/12 (67%)
Mortality (%)	14 (37.8%)
APACH II score	19 (15–27)
SOFA score	9 (5.5–11.5)
**ARDS (n=19)**	
Age	70 (60–80)
Male gender (n, %)	12/7 (63%)
Mortality (%)	7 (36.8%)
APACH II score	25 (22–28)
SOFA score	9 (7.5–12)
PaO_2: FiO_2 ratio	126.3 (91.5–177.7)
**Burns (n=35)**	
Age	56 (41–77)
Male gender (n, %)	18/17 (51%)
Mortality (%)	5 (14%)
SOFA score	3 (0.75–6)
% TBSA	30 (33–52)
BI	25 (30–35)
PBI	82 (70.5–98)

Table S1. Characteristic features and clinical course of patients with sepsis, ARDS and burns. %TBSA, percentage of the total body surface area; APACHE, Acute Physiology and Chronic Health Evaluation; BI, burn index; PBI, prognostic burn index; SOFA, Sequential Organ Failure Assessment. PaO_2, partial pressure of arterial oxygen; FiO_2, percentage of inspired oxygen. Data are shown as group number or median (interquartile range). Missing values were not included in the table.

TABLE S2

Case	Age/Gender	Complications	Anti-viral drugs combined with TCZ	Requiring O2 conc. and laboratory findings before TCZ	Clinical features and laboratory findings after TCZ	Patient condition
1	72/M	NTM,<br>COPD,<br>Lower pharyngeal cancer	F+C	O2 3L/min,<br>Lym 175/μL, CRP 8.62 mg/dL,<br>Ferritin 153.1 ng/mL	Day 5: CRP 0.30, Lym 470<br>Day 8: O2 2L/min<br>Day 21: CRP 0.05, Lym 970, Ferritin 77.2<br>Day 25: SARS-CoV-2 (-)<br>Day 27: Discharge with HOT	Cure
2	41/M		F+C	O2 2~3 L/min,<br>Lym 530/μL, CRP 13.51 mg/dL,<br>Ferritin 1522 ng/mL	Day 5: O2 free<br>Day 5: CRP 1.07, Lym 1150, Ferritin 889.4<br>Day 11: CRP 0.10, Lym 1580, Ferritin 652.9<br>Day 11: SARS-CoV-2 (-)<br>Day 12: Discharge	Cure
3	63/M	HT	F+C	O2 3~5 L/min,<br>Lym 1280/μL, CRP 11.53 mg/dL,<br>Ferritin 1531.8 ng/mL	Day 3: CRP 1.98, Lym 990, Ferritin 943.9<br>Day 7: O2 free<br>Day 10: CRP 0.12, Lym 1664, Ferritin 592.5<br>Day 15: SARS-CoV-2 (-)<br>Day 16: Discharge	Cure
4	68/M	DM	F+C	O2 2~6 L/min,<br>Lym 999/μL, CRP 2.33 mg/dL,<br>Ferritin 880.6 ng/mL	Day 5: O2 free<br>Day 7: CRP 0.17, Lym 1400, Ferritin 828.5<br>Day 13: CRP 0.06, Lym 1350, ferritin 1120.8<br>Day 14: SARS-CoV-2 (-)<br>Day 15: Discharge	Cure
5	71/M	DM	L/R+C	Under artificial ventilation (FiO2 0.35~0.8),<br>Lym 2130/μL, CRP 7.47 mg/dL,<br>Ferritin 4383.4 ng/mL	Day 4: CRP 1.13, Lym 1010<br>Day 12: Under artificial ventilation (FiO2 0.3)<br>Day 25: SARS-CoV-2 (-)	Improvement
6	79M		F+C	O2 4L/min,<br>Lym 860/μL, CRP 8.57 mg/dL,<br>Ferritin 1110.7 ng/mL	Day 2: Intubation and transfer to other hospital<br>Day 2: CRP 3.9, Lym 660, Ferritin 1505.6<br>Day 13: Extubation and transfer to our hospital<br>Day 13: CRP 0.46, Lym 581, Ferritin 800.4<br>Day 15: O2 free	Recovery from deterioration
7	48/M	DM<br>HT<br>Obesity<br>SAS	F+C	O2 6L/min,<br>Lym 82/μL 0, CRP 3.78 mg/dL,<br>Ferritin 3355.4 ng/mL	Day 1: Intubation and transfer to other hospital<br>Day 9: Extubation and transfer to our hospital<br>Day 13: CRP 0.12, Lym 278, Ferritin 3700.9 followed by mPSL pulse	Recovery from deterioration

Table S2. Characteristic features and clinical course of patients with COVID-19 treated with tocilizumab. TCZ: tocilizumab; DM: diabetes mellitus; HT: hypertension; NTM: non-tuberculous mycobacteriosis; COPD: chronic obstructive pulmonary diseases; F: favipiravir; C: ciclesonide; L/Rlopinavir/ritonavir.
